# Unlocking the Interaction Mechanism of CNTs and C‐S‐H on Enhancing Elastic and Viscoelastic Properties of Alite Paste

**DOI:** 10.1002/advs.202505876

**Published:** 2025-07-17

**Authors:** Xi Chen, Jiseul Park, Weiyi Ji, Yujie Huang, Jian‐Xin Lu, Zhangli Hu, Chi Sun Poon

**Affiliations:** ^1^ Department of Civil and Environmental Engineering and Research Centre for Resources Engineering Towards Carbon Neutrality The Hong Kong Polytechnic University Hung Hom Kowloon Hong Kong China; ^2^ Department of Architecture and Architectural Engineering Seoul National University 1 Gwanak‐ro, Gwanak‐gu Seoul 08826 Republic of Korea; ^3^ School of Materials Science and Engineering Jiangsu Key Laboratory of Construction Materials Southeast University Nanjing 210098 China

**Keywords:** carbon nanotubes, molecular dynamics simulation, nucleation effect, tricalcium silicate, viscoelastic

## Abstract

Carbon nanotubes (CNTs) have demonstrated potential in enhancing the elasticity and viscoelasticity of cementitious materials, yet the mechanisms underlying their influence on hydrates remain unclear. This study employs experimental techniques and molecular dynamics simulations to elucidate the impact of CNTs on alite hydration, spanning initial stages to the hardened state. Results show that CNTs exhibit a weak nucleation effect, which is thus not the primary factor contributing to the enhancement of mechanical properties. Instead, improvements stem from refining the calcium silicate hydrate (C‐S‐H) gel structure and optimizing the spatial distribution of calcium hydroxide (CH) throughout hydration. The CNT‐mediated modulation of Ca^2+^ distributions strengthens C‐S‐H cohesion, refines pore size, and promotes the formation of high‐density C‐S‐H, thereby improving elastic properties. Additionally, the influence of CNTs on shear deformation and particle orientation among C‐S‐H particles is highlighted for viscoelastic properties. These insights redefine CNT contributions, emphasizing their role in long‐term ionic and structural tuning, and provide a foundation for designing advanced nanofiber‐cement composites with superior mechanical performance.

## Graphical abstract

This study explores carbon nanotubes’ (CNTs) role in enhancing alite paste’ elasticity and viscoelasticity. Experimental and simulation results reveal that CNTs weakly influence nucleation but significantly refine C‐S‐H gel structure and calcium hydroxide distribution. By modulating Ca^2+^ distributions, CNTs strengthen cohesion, reduce pore size, and influence the shear deformation and particle orientation, offering new insights for designing advanced nanofiber‐cement composites.

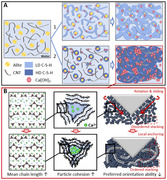

## 1 Introduction

Cement is by far the most widely used building material in the world. With the ongoing urbanization in developing countries and the continuous growth of the global population, cement is poised to remain an indispensable man‐made material for the foreseeable future.^[^ ^1^ ^]^ However, concrete made of hydrated cement as a binding material is typically brittle and is prone to cracking under tensile stress. The resulting durability problems have always been a key concern in the design of engineering structures, especially as the emergence of ultrahigh, large‐span, and lightweight infrastructures has placed higher demands on the superior performance of cement‐based materials.^[^ ^2^ ^]^

The advent of nanoengineering has spurred extensive research into optimizing the properties of cementitious materials through the use of nanomaterials.^[^ ^3^, ^4^, ^5^ ^]^ For instance, the incorporation of nano‐alumina^[^ ^6^ ^]^ and nano‐silica^[^ ^7^ ^]^ yielded denser hydration products, thereby enhancing the strength and elastic modulus of concrete. Moreover, graphene^[^ ^8^, ^9^ ^]^ and carbon nanotubes^[^ ^10^, ^11^ ^]^ have been shown to significantly improve the mechanical properties of cementitious materials at very low concentrations, owing to their high specific surface area and exceptional mechanical properties. This has led to numerous studies on grafted nanocarbon additives, focusing on enhancing the bond between nanomaterials and the cementitious matrix by incorporating functional groups such as hydroxyl or carboxyl groups.^[^ ^12^, ^13^, ^14^ ^]^

Considering both performance and cost, multi‐walled carbon nanotubes (CNTs) have emerged as the most commonly used and extensively studied nanocarbon additives for toughening cementitious materials.^[^ ^15^ ^]^ Studies have demonstrated that incorporating less than 0.5 wt.% of multi‐walled CNTs can increase the flexural strength, compressive strength, and elastic modulus of plain cement paste by up to 269%, 58%, and 59%, respectively.^[^ ^16^, ^17^, ^18^ ^]^ However, the extent of property enhancement in cement‐based materials by CNTs remains controversial due to variables such as type, content, dispersion degree, and experimental methodologies.^[^ ^15^, ^19^, ^20^, ^21^ ^]^

Researchers have proposed several mechanisms on the enhanced mechanical properties of cementitious materials with CNTs: i) Crack‐bridging effect, where CNTs effectively bridge microcracks;^[^ ^22^ ^]^ ii) Filling effect, where CNTs refine the pore structure and increase the proportion of high‐density calcium silicate hydrate (HD C‐S‐H);^[^ ^23^ ^]^ iii) Nucleation effect, where CNTs, due to their high specific surface area, absorb calcium ions and provide additional nucleation sites during cement hydration, thereby accelerating the hydration rate;^[^ ^15^, ^24^ ^]^ iv) Enhancement of C‐S‐H polymerization and extension of the average length of silicate chains.^[^ ^25^ ^]^

Despite these insights, misunderstandings and controversies persist regarding these mechanisms. Some researchers have attributed the strength increase in cement‐based materials directly to CNT nucleation, suggesting that the elevated HD C‐S‐H ratio in hydration products resulted from the provision of nucleation sites.^[^ ^15^, ^26^ ^]^ Conversely, experimental evidence indicated that CNTs did not provide strong nucleation sites.^[^ ^27^ ^]^ Thus, beyond the acknowledged bridging effect, the contributions of other mechanisms to concrete strength enhancement remain unclear. Furthermore, whether the high percentage of HD C‐S‐H arises from numerous C‐S‐H particles attached to CNT surfaces or from CNT‐induced alterations in C‐S‐H particle interactions requires further investigation. Additionally, these mechanisms have primarily been explored focusing on compressive, tensile, and flexural strength enhancements,^[^ ^28^, ^29^, ^30^, ^31^ ^]^ with a lack of systematic studies on how CNTs influence the elasticity and viscoelasticity of cementitious materials, such as elastic modulus and creep behavior.

To address the unresolved questions, we investigated the effects of CNTs on an alite (main phase in cement) paste from early hydration to 28‐day hardening. Utilizing heat flow curves, transmission electron microscopy (TEM), inductively coupled plasma‐optical emission spectrometry (ICP‐OES), and molecular dynamics simulations (MD), we refuted the hypothesis that CNT nucleation was the primary reason for enhanced HD C‐S‐H occupancy and clarified the role and mechanism of CNTs in enhancing inter‐particle cohesion among C‐S‐H particles. Additionally, we provided the first visual evidence of CNTs' influence on calcium hydroxide (CH) distribution using confocal Raman microspectroscopy (CRM). Furthermore, we revealed the mechanisms of how CNTs improve elasticity and viscoelasticity of alite‐CNT composites by integrating indentation, atomic force microscopy (AFM), and nuclear magnetic resonance (NMR) experimental techniques, alongside analyses of the stacking orientation of hydration products and the molecular structure within C‐S‐H particles.

Our multi‐scale study on the interaction of CNTs and alite elucidates the current misconceptions, emphasizes the importance of nanomaterials for cement cohesion and particle stacking orientation of hydration products, and provides a theoretical basis for the future development of nanoengineering in cementitious materials.

## 2 Results

The study of the interaction mechanism between CNTs and cement‐based materials is a well‐established field. Given the current fragmented and contradictory research status, we conducted experiments and simulations to provide clarity. We selected a pure clinker phase in cement, alite, as a substitute for cement and designed four sample groups with varying CNT contents (0, 0.1%, 0.2%, and 0.3%). The reference group is denoted as R, while T1 to T3 represent groups with increasing CNT dosages.

### 2.1 Early Hydration Effect

**Figure 1:**
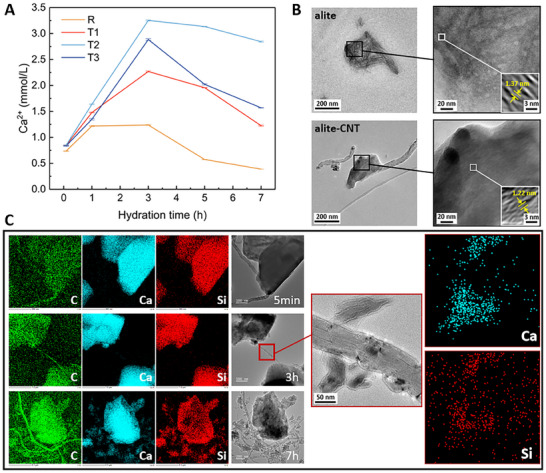
Early hydration effect analysis of alite‐CNT composites. A) Based on the acceleration period, five time points were selected to determine the calcium ion concentration in the pore solutions with a water‐to‐alite ratio of 100. B) Morphologies of hydration products at 7 h. C) Corresponding to the hydration time of (A), elemental analysis was carried out on carbon (green), calcium (cyan), and silicon (red), respectively. To more clearly compare the distribution characteristics of Ca and Si, the period of the highest ion concentration in the pore solution (3 h) was selected to amplify the elements' distribution at the local nucleation site of CNT.

Initially, we examined the effects of CNTs on early hydration. The isothermal calorimetry results revealed that the heat flow peaks for T1, T2, and T3 groups occurred 0.07, 0.16, and 0.05 h earlier than the reference group, respectively (Figure S1, Supporting Information). This trend suggests that CNTs slightly enhanced the formation and precipitation of hydration products, thereby accelerating the hydration of alite. Notably, the acceleration effect was the weakest in T3, likely due to CNT agglomeration at higher dosages, which limited their efficiency within the matrix. During the acceleration phase, we characterized the alite pore solutions using ICP‐OES (**Figure** **1**A). The calcium ion concentration peaked at 3 h across all groups. The addition of CNTs significantly increased Ca^2+^ levels in the pore solutions compared to the reference, indicating that CNTs enhanced alite dissolution.

To elucidate the mechanism behind the influence of CNTs on alite dissolution and precipitation, we employed field emission TEM to observe the morphologies and elemental distributions of alite‐CNT composites at 5 min, 3 h, and 7 h. Several nucleation sites were identified on the CNT surfaces (Figure S2A, Supporting Information). However, the sparse distribution of these sites relative to the high specific surface area of CNTs suggests a limited seeding effect, consistent with previous studies.^[^ ^27^ ^]^ Surprisingly, we find that the incorporation of CNTs led to a more densely packed arrangement of the nucleation sites compared to the alite paste (Figure 1B). Beyond the reduction of gel pore size, the local interlayer spacing of C‐S‐H, as determined by Fourier transform calculations from high‐resolution imaging, also indicates the improvement in interlayer cohesion attributable to CNTs.

Additionally, we conducted large‐area mapping analyses of calcium and silicon elements at local nucleation sites at the three specified hydration time periods (Figure 1C). After taking into account the carbon contents of the sample holders, the carbon distribution allowed us to locate CNTs effectively. After 5 min, dislocations appeared at the edges of alite particles, marking the onset of dissolution.^[^ ^32^ ^]^ At this stage, calcium and silicon were evenly distributed around the alite. As hydration progressed to 3 and 7 h, silicon remained uniformly dispersed in regions other than hydration products, while calcium became more concentrated around the CNT surface. For example, in a localized zoomed‐in image at 3 h, calcium was observed to accumulate more at nucleation sites and on CNT surfaces compared to silicon. This indicates that CNTs exhibited a specific adsorption effect on calcium ions in the pore solution. Therefore, although CNTs promoted the further dissolution of alite by altering the ionic equilibrium in the solution, leading to an increase in calcium ion concentration (Figure 1A), most hydration products still nucleated on the clinker surfaces (Figure S2A, Supporting Information). Consequently, CNTs provided limited assistance in expanding the growth space for C‐S‐H, resulting in a minimal acceleration of the early hydration reaction rate. Simultaneously, the denser hydration products induced by CNTs may impede the further dissolution of encapsulated unhydrated alite during the induction period, which could also account for the inconspicuous acceleration of hydration.

Moreover, we also performed multi‐point elemental analysis of nucleation sites surrounding CNTs (Figure S2B, Supporting Information). Our findings revealed that the calcium‐to‐silicon ratio (Ca/Si) of hydration products farther from the CNT surface ranged from ≈1.31 to 1.79, consistent with the typical range for C‐S‐H.^[^ ^32^ ^]^ In contrast, hydration products near the CNT surface exhibited higher Ca/Si ratios, predominantly between 1.96 and 2.13. Based on related studies,^[^ ^24^, ^32^ ^]^ the elevated Ca/Si ratio near the CNT surface indicated the formation of calcium‐rich hydration products, potentially a mixture of C‐S‐H and a transitional phase resembling CH. We hypothesize that, as hydration progresses, these products may contribute to the growth of more hydration products in later stages. In particular, areas near the CNT surface with a Ca/Si ratio greater than 1.96 may increase the proportion of the CH phase in the final hydration products. This hypothesis will be further explored in conjunction with subsequent experimental results.

### 2.2 Pores and Hydration Products

**Figure 2:**
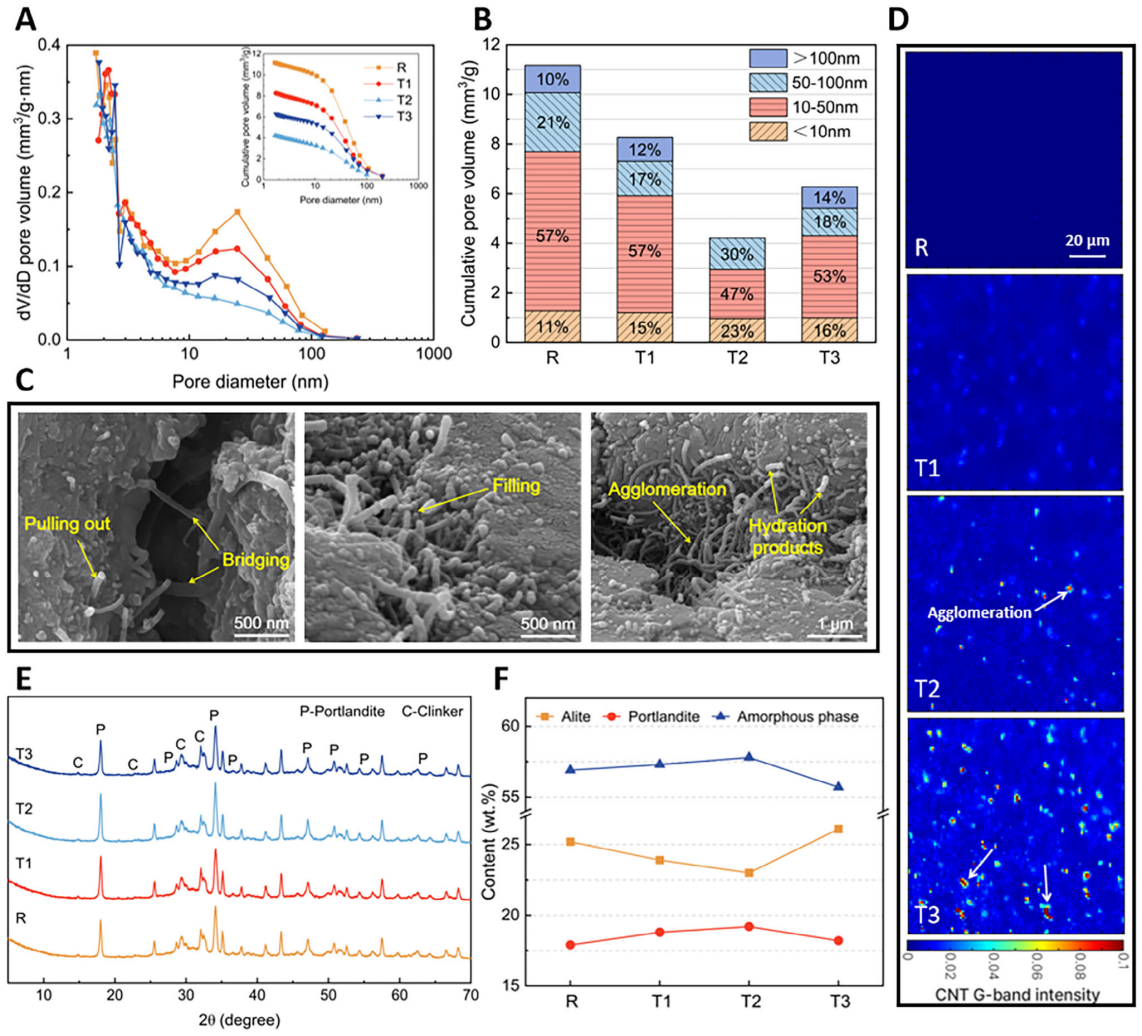
Pores and hydration products analysis of alite‐CNT composites. A) Pore size distributions of alite‐CNT composites, with the corresponding cumulative pore volume curves in the upper right corner. Based on the results of (A), different pore size proportions were calculated in B). Morphologies of T3 were obtained in C), and some nucleation sites (hydration products) can be observed. D) shows the dispersion of CNTs within the pastes. The brighter the dots, the higher the agglomeration of CNTs. XRD E) and the quantitative analysis results F) indicate that the appropriate amount (less than 0.3%) of CNT had a promoting effect on the hydration of alite.

For the hardened alite‐CNT pastes, pore size distributions were initially assessed using the nitrogen adsorption method (**Figure** **2**A). The addition of CNTs was found to reduce the cumulative pore volume, with the most pronounced effect observed at a CNT content of 0.2%, where the pore volume decreased from 11.2% to 4.2%. However, excessive CNT content (0.3%) led to agglomerations that may form additional pores. For quantitative analysis, we categorized the pore sizes into four groups: large capillary pores (>100 nm), medium capillary pores (50–100 nm), small capillary pores (10–50 nm), and gel pores (<10 nm) (Figure 2B).^[^ ^33^, ^34^ ^]^ At 0.1% CNT, the pore size distribution was similar to the reference. As the CNT content increased, large capillary pores disappeared in T2, and the proportion of gel pores increased, indicating a refining effect on pore size. Conversely, at 0.3% CNT, while the pore size was refined, a certain percentage of large capillary pores reappeared. To visually assess the impact of CNTs on the pastes, field emission scanning electron microscopy (FESEM) was employed to observe at T3 as an example (Figure 2C). Typical crack‐bridging and pull‐out effects were observed at larger cracks. Voids were noted where CNTs agglomerated, and for smaller gaps, CNTs facilitated the filling by carrying some hydrated products. These morphologies were aligned with previous studies.^[^ ^35^, ^36^, ^37^ ^]^

Spatial distributions of phases in the alite‐CNT composites were obtained using confocal Raman microspectroscopy (CRM) (Figure 2D). This method was chosen because CRM can observe a larger area and is representative compared to traditional methods such as TEM or FESEM. More importantly, CRM is capable of analyzing the dispersion of CNTs within the hardened pastes in situ, which is more convincing than judging the dispersion only from the CNT suspension.^[^ ^38^, ^39^ ^]^ The Raman peak intensity is proportional to the number of analyte molecules in the interaction volume.^[^ ^40^ ^]^ Therefore, warmer colors (e.g., red) indicate higher concentrations, and cooler colors (e.g., blue) represent lower concentrations or the absence of CNTs. In the reference sample (R), no G‐band was detected. At lower CNT concentrations (T1 and T2), CNTs were relatively well dispersed, although small aggregates started to form in T2 (marked by arrows). However, in T3, significant agglomeration was evident, with larger clusters of CNTs appearing as bright red regions. These observations highlight the reduced dispersion efficiency of CNTs at higher concentrations, consistent with previous studies reporting CNT bundle sizes of several micrometers in poorly dispersed systems.^[^ ^41^, ^42^ ^]^

Figures S3 and S4 (Supporting Information) present the spatial distribution maps of each phase obtained from the linear coefficient of the basis spectrum. In Figure S4A (Supporting Information), the color bar scales were standardized across samples to enable a direct comparison of the relative volume fractions of each phase. The hydration products—Ca(OH)₂ and C‐S‐H—were preferentially precipitated around the C_3_S clinker particles and filled the space between clinkers. The C‐S‐H intensity was significantly higher in T1 compared to R, indicating an enhancement in C‐S‐H precipitation. Furthermore, the pore areas between clinkers were more densely filled with C‐S‐H in T1 than R, as shown in the marked circles, suggesting that CNTs facilitate the densification of hydration products. In contrast, the extensive and intense C_3_S regions observed in T3 suggest a higher fraction of unreacted C_3_S, indicating that excessive CNT dosage may hinder the hydration process of C_3_S.

In addition, we conducted further analysis on the distribution of C_3_S and Ca(OH)_2_ in Figure S4B (Supporting Information). We extracted and superimposed the areas of C_3_S and Ca(OH)_2_ from four sample groups to compare the impact of CNTs on Ca(OH)_2_ distribution, the black area represented the region containing only C‐S‐H. Note that we ignored variations in intensity at different locations and only extracted the distribution areas of the two phases, which did not represent the volume occupied by each phase. We found that in the reference group, the distributions of these two phases were nearly completely overlapping. Interestingly, as CNT content increased, Ca(OH)_2_ began to grow in localized areas distant from C_3_S, particularly in the T2 group (circled area). Additionally, as depicted in Figure 2D, although some agglomeration occurred, CNT intensity was consistently detected throughout the regions in groups T1 to T3. Thus, we can deduce that the uniform distribution of CNTs may contribute to the free growth of CH. Because a previous work^[^ ^10^ ^]^ demonstrated that CNTs increased the orientation index of CH, thereby providing more free space for CH growth. Additionally, another study^[^ ^27^ ^]^ investigated the alite pore solution with CNTs and observed lamellar CH growing around the CNTs. This phenomenon aligns with our hypothesis in Section 2.1, suggesting that calcium ions enriched around CNTs may increase the proportion of CH. Also, the hypothesis previously observed in CT experiment, that the hydration products around CNT aggregates were CH, aligns with our conjecture.^[^ ^11^ ^]^

To quantitatively analyze CNT effects on alite hydration degree, crystalline phases within pastes were measured using X‐ray diffraction (XRD) (Figure 2E,F). At 0.1% and 0.2% CNT content, alite content decreased compared to the reference, indicating a higher degree of hydration. Additionally, compared to R, we observed varying degrees of increase in portlandite content across groups T1 to T3, aligning with CRM observations.

### 2.3 Interparticle Interactions of C‐S‐H

**Figure 3:**
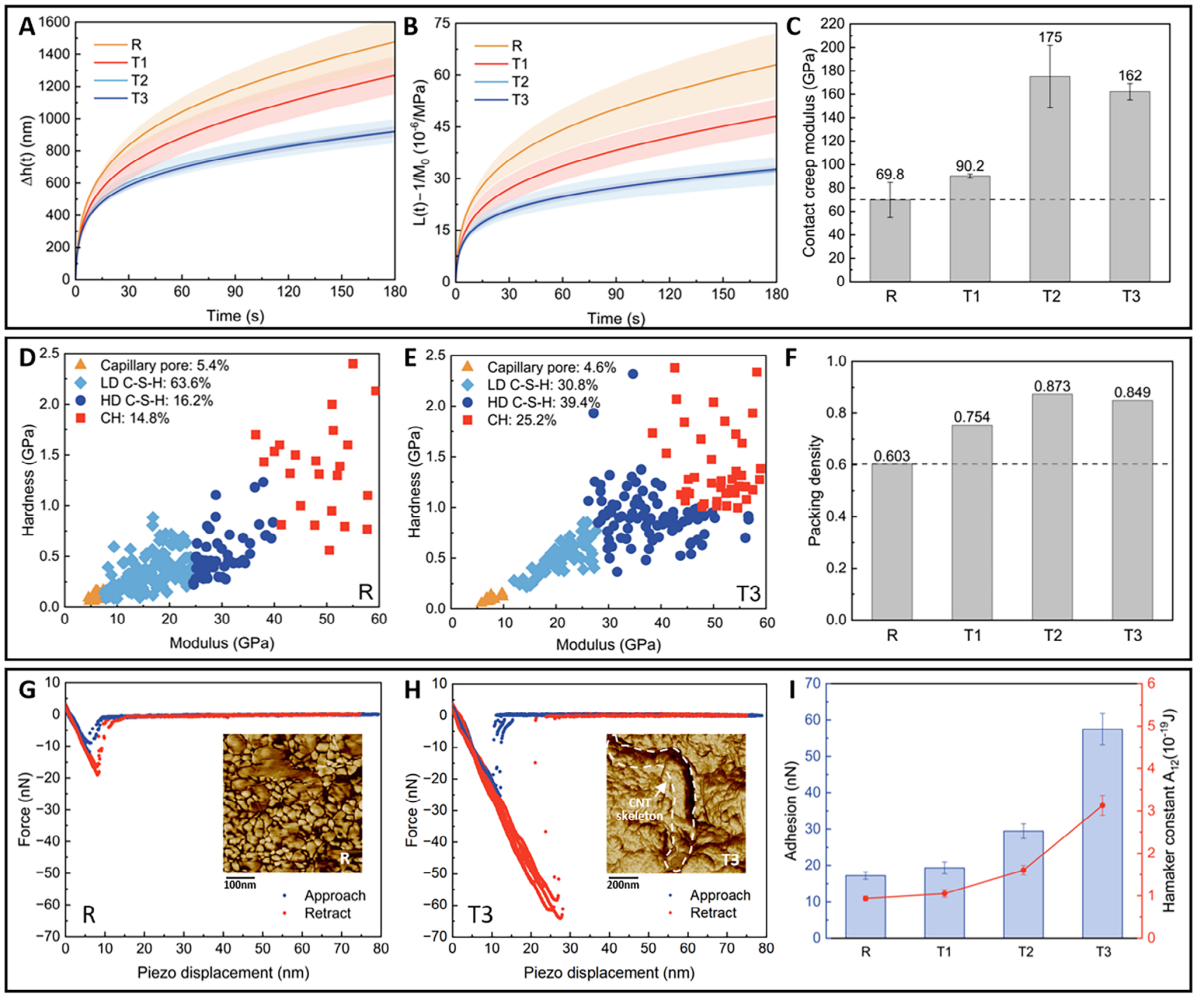
Interparticle interactions of C‐S‐H in alite‐CNT composites. A) and B) are the variation curves of microindentation depth and creep compliance with load‐holding time. After the fitting process, the contact creep modulus was calculated in C). D) and E) are the cluster analysis of pastes with 0% and 0.3% CNTs tested by nanoindentation. Also, we calculated the packing density to quantitatively analyze the stacking of hydration products in F). Morphologies and force‐displacement curves were obtained using atomic force microscopy in G) and H). Adhesion force and the Hamaker constant were also calculated in I) based on the curves.

Given that CNTs promoted the hydration of alite and possessed a unique nanofibrous structure, we were intrigued by the behavior of alite‐CNT composites under stress, particularly focusing on the primary hydration phase, C‐S‐H. This was related to the interaction of CNTs with C‐S‐H and the impact of CNT doping on interactions between C‐S‐H particles. We first examined the long‐term creep properties of alite‐CNT pastes using a microindentation test with a high load (1.5 N) and an extended holding stage (180 s), a method validated in several studies.^[^ ^43^, ^44^, ^45^ ^]^ We calculated the variation in indentation depth and creep compliance over the load‐holding period (**Figure** **3**A,B). Due to the non‐homogeneity of the hardened pastes, the curves exhibited some fluctuation. Results indicated that both indentation depth and creep compliance decreased with increasing CNT content compared to the reference, suggesting that CNTs can mitigate the creep of hardened pastes. For quantitative analysis, we fitted the curves to determine the contact creep modulus (Figure 3C). Compared to the reference, the creep modulus increased by 28.9%, 150.3%, and 131.7% for T1, T2, and T3, respectively. The reduced growth in T3 compared to T2 may be attributed to the adverse effects of a larger number of capillary pores.

Additionally, we employed a nanoindentation test with a low load (2000 µN) and a short holding stage (5 s) to investigate the proportion and packing density of hydration products. The choice of smaller loads ensured that the affected material area under the indenter was minimized, allowing for better differentiation of the various phases.^[^ ^46^ ^]^ We investigated four hydration product phases: capillary pores, LD C‐S‐H, HD C‐S‐H, and CH. Clustering analysis revealed modulus ranges of <9.7 GPa for capillary pores and >36.4 GPa for CH across all four sample groups. The modulus values of LD C‐S‐H and HD C‐S‐H range from 12.3–26.6 GPa and 27.2–38.5 GPa, respectively. These modulus values align with those reported in a prior study.^[^ ^46^ ^]^ Taking samples R and T3 as examples, clustering analysis demonstrated that CNT incorporation markedly enhanced the proportions of HD C‐S‐H and CH. To quantify LD C‐S‐H and HD C‐S‐H, we combined nanoindentation clustering results with XRD data (Figure 2F) to determine the precise mass fractions of LD C‐S‐H and HD C‐S‐H across four sample groups (Figure S5, Supporting Information). At a CNT content of 0.2 wt.% (T2), the content of HD C‐S‐H reached its maximum, reflecting optimized C‐S‐H packing induced by CNTs, which corroborated the packing density trends observed in Figure 3F.

To quantitatively analyze the cohesion between C‐S‐H particles before and after packing density enhancement, we used atomic force microscopy (AFM) to measure the morphology and force‐displacement curves of alite‐CNT composites (Figure 3G–I). Here, we implemented a previously reported method,^[^ ^80^ ^]^ limiting the maximum compressive force of the force‐displacement curve to 3 nN (with penetration under 2 nm) to reduce sample deformation and protect the tip. The surface roughness of the samples, ranging from 6 to 13 nm, was deemed suitable for surface force measurements.^[^ ^47^ ^]^ Comparing the morphologies of R and T3, we observed that CNT addition significantly enhanced densification between C‐S‐H particles, exhibiting a nanoskeleton‐like functionality bonded to surrounding hydration products. Adhesion force and the Hamaker constant were used to characterize the inter‐particle interactions.^[^ ^47^ ^]^ The results confirmed that CNTs enhanced cohesion between C‐S‐H particles. However, for T3, the improvement was greater than the results indicated by the indentation test. This discrepancy may be due to the AFM test minimizing or avoiding the influence of surrounding pores, thus highlighting the positive effect of higher CNT content on cohesion enhancement between C‐S‐H particles. Previous studies have also highlighted that porosity significantly affected the Young's modulus and creep of C‐S‐H.^[^ ^48^ ^]^

### 2.4 Molecular Structure of C‐S‐H

**Figure 4:**
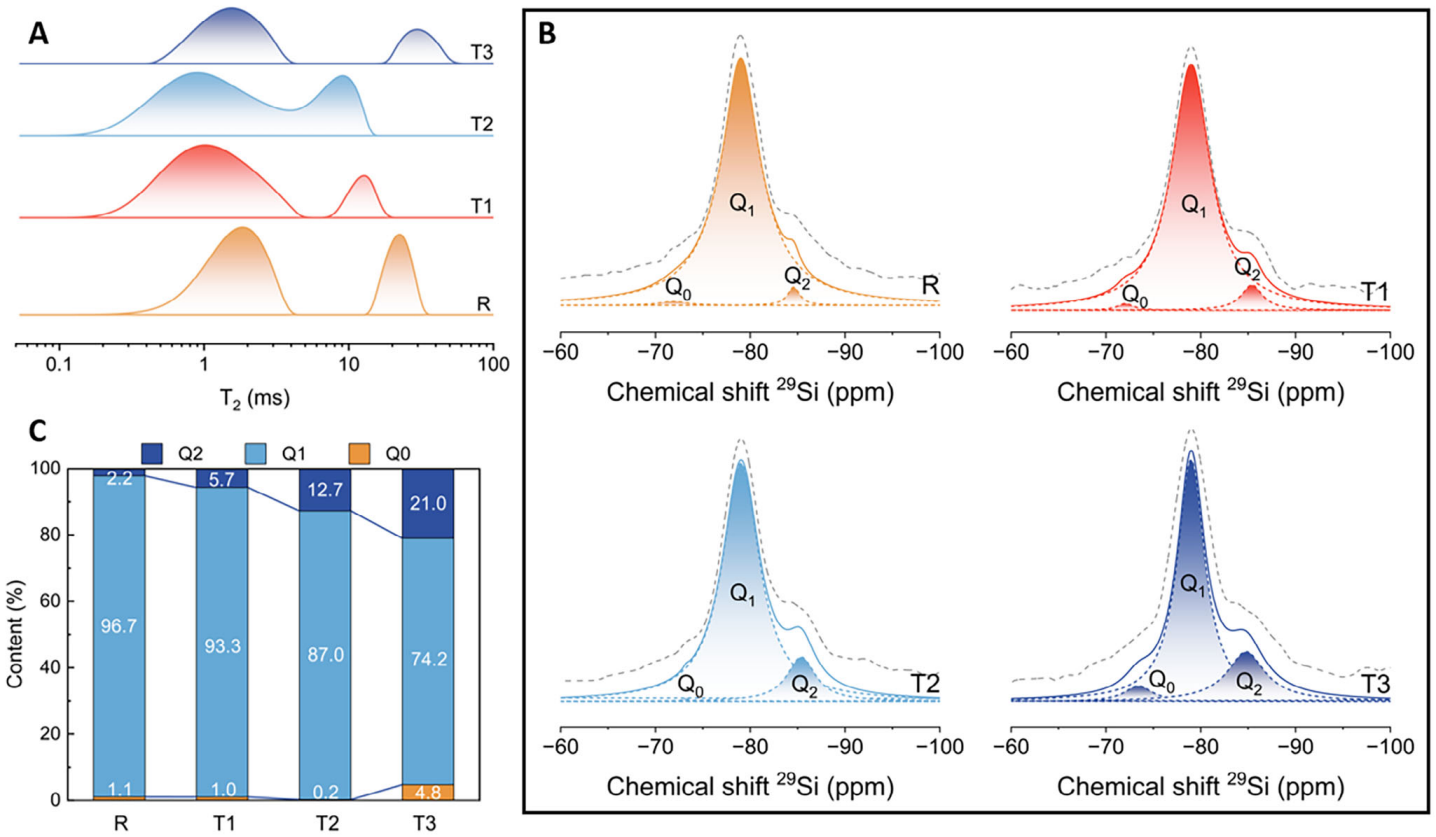
Molecular structure analysis of C‐S‐H in alite‐CNT composites. A) The inverted curves from ^1^H NMR spectra of alite‐CNT composites. B) Deconvolution analysis results of ^29^Si NMR spectra. Based on (B), the Q^n^ proportions were calculated in C).

Next, we turned our attention to the molecular structure of the C‐S‐H particles themselves. While we previously assessed the pore size distribution of alite‐CNT composites using the N_2_ adsorption method (Figure 2A,B), the changes in smaller‐sized pores, such as fine gel pores, remained unclear. To address this, we employed a ^1^H NMR spectrometer to analyze the fine pores of the hardened pastes (**Figure** **4**A). Two distinct peaks were identified at T2 values <2 and >9 ms, corresponding to gel pores and capillary pores, respectively, as supported by related studies.^[^ ^33^, ^49^ ^]^ The pore size trends observed were consistent with those obtained via the N_2_ adsorption method. The second peaks (>9 ms) for T1 and T2 shifted leftward compared to the reference. At the same time, T3 exhibited a rightward shift, indicating an initial decrease followed by an increase in the size of capillary pores. We focused on the first peak, representing gel pores (< 2 ms). It is important to clarify that the interlayer space, while not a pore in the traditional sense, is part of the molecular structure of a single C‐S‐H particle. These interlayer spaces can exchange water with gel pores, thus being considered fine gel pores in terms of size.^[^ ^50^ ^]^ We found that incorporating 0.1%–0.2% CNTs shifted the first peak leftward compared to the reference (from 1.75 to 1 ms and 0.87 ms), with a broadened peak distribution, indicating refined gel pore sizes. This suggests that the connected interlayer spacing may also be influenced by this refinement. These findings align with TEM observations (Figure 1B). Although excess CNTs (0.3%) enlarged capillary pores, the gel pore peaks still shifted to the left by 0.23 ms compared to the reference.

Following our investigation of CNT effects on C‐S‐H interlayer spacing, we used a ^29^Si NMR spectrometer to analyze the degree of polymerization within the C‐S‐H layers (Figure 4B,C). Based on the deconvolution curves, we calculated the proportion of Q^n^ (*n* = 0, 1, and 2), which represents the number of bridging oxygens tetrahedrally coordinated with the silicon atom.^[^ ^51^ ^]^ For hydrated alite, there were three peaks located at ≈−73, −79, and −85 ppm, corresponding to Q^0^, Q^1^, and Q^2^.^[^ ^52^ ^]^ After adding 0.1% and 0.2% CNTs, the proportion of Q^0^ gradually decreased and nearly disappeared compared to the reference, indicating that CNT promoted the hydration of alite. However, when the content increased to 0.3%, the Q^0^ ratio increased significantly, suggesting the inhibited hydration process by excessive CNTs. The Q^2^ content of T1 to T3 showed a steady rise. We also calculated the mean chain lengths of 2.05, 2.12, 2.29, and 2.57 for R, T1, T2, and T3, respectively. Longer mean chain lengths indicate a higher degree of polymerization, demonstrating that CNTs can enhance the degree of C‐S‐H polymerization.

### 2.5 Molecular Dynamics Simulation

**Figure 5:**
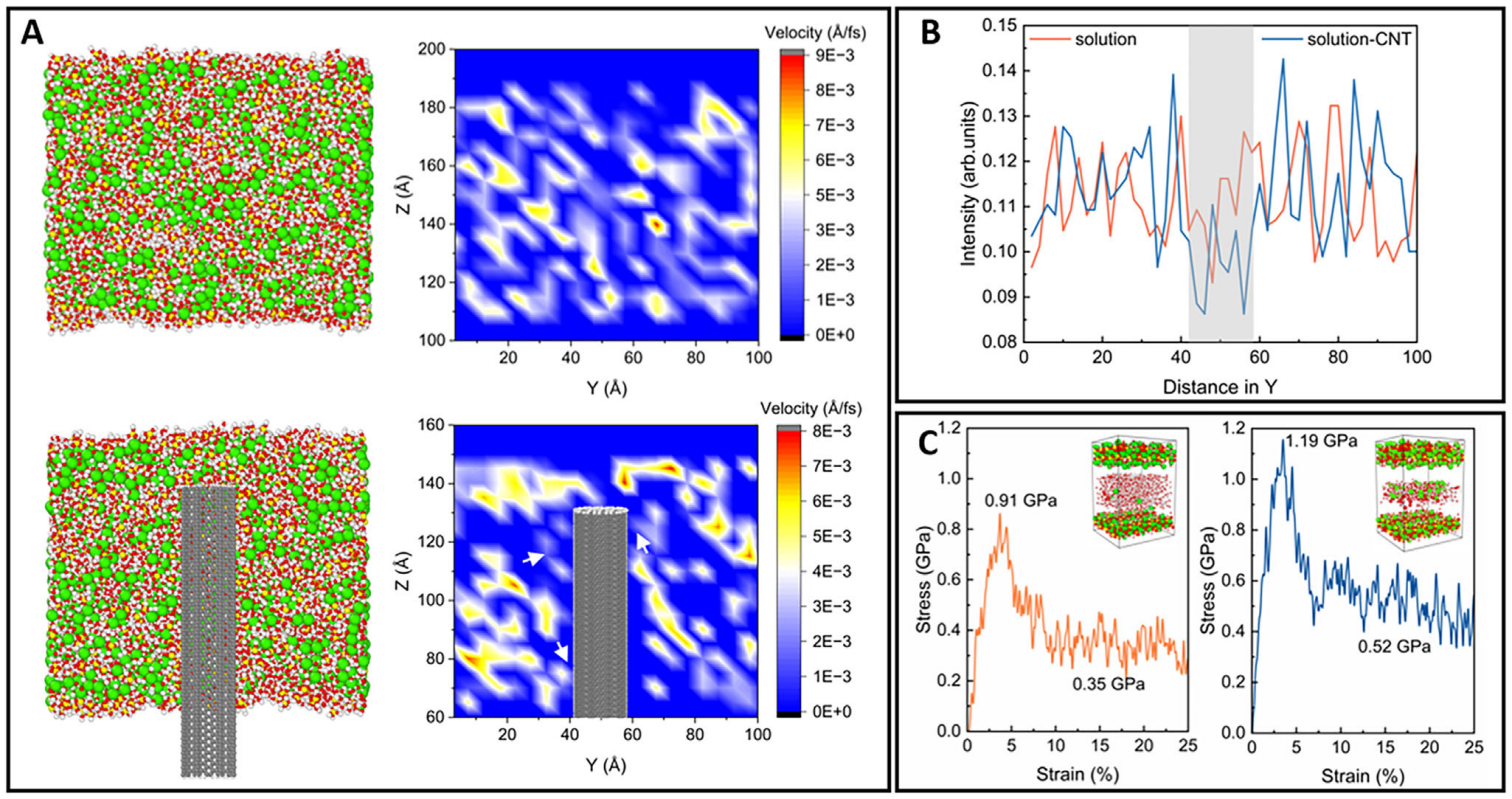
Molecular dynamics simulations of alite‐CNT composites on early hydration and creep behaviors. A) Simulation of interactions between CNT and calcium ions in solutions during the dissolution phase of alite. The velocity distributions of calcium ions without CNT and with CNT were output respectively, and the location of CNT was marked in the graph. We also output the density distribution of Ca^2+^ along the Y direction in B), where the shaded region is the position of the CNT. C) For creep simulation under shear load, the two models represent the interaction of C‐S‐H in hardened alite paste (left) and alite‐CNT paste (right), respectively. Calcium (green), silicon (yellow), oxygen (red), hydrogen (white), and carbon (gray).

Based on the experimental results, several aspects remain unresolved. The first is the adsorption of calcium ions by CNTs (Figure 1C). The interaction mechanism between Ca^2+^ ions and CNTs is not well understood. Additionally, the mechanism of CNTs on the creep behaviors of C‐S‐H is unclear. To address these issues, we employed molecular dynamics simulations to analyze these phenomena at the atomic scale. For ion adsorption, we modeled two pore solutions: one without a CNT and one with a CNT (Figure S7A, Supporting Information). By comparing the Ca^2+^ velocity distribution diagrams, we observed that the CNT slightly hindered the movement of calcium ions (marked by arrows), indicating a degree of adsorbability (**Figure** **5**A). Furthermore, we analyzed the density distribution of calcium ions along the Y‐direction in both models (Figure 5B). Without a CNT in the solution, the Ca^2+^ distribution was more uniform. However, after inserting the CNT, there was a slight increase in ion concentration on the CNT surface due to the electrostatic attraction between calcium ions and the CNT, consistent with the phenomenon observed in Figure 1C.

Regarding the improvement of creep by CNTs, we constructed two comparative models (Figure 5C). The pore sizes were based on experimental results from nitrogen adsorption (Figure 2A) and ^1^H NMR (Figure 4A), focusing on various sizes of fine gel pores. For the ionic concentration of the pore solution, we considered the calcium ion trends obtained by ICP‐OES (Figure 1A). The first model represented alite paste without CNTs, featuring larger gel pores and fewer calcium ions. The second model represented alite‐CNT paste, with smaller gel pores and more calcium ions (Figure S7B, Supporting Information). Since creep occurs under shear, we generated stress‐strain curves for both models under shear loading. The trends were similar: after reaching maximum shear stress, the stress fluctuated around a mean value as the strain increased (Figure 5C). The maximum shear stresses for the two models were 0.91 and 1.19 GPa, respectively, with subsequent average shear stresses of 0.35 and 0.52 GPa. These results indicate that the interlayer slip resistance of C‐S‐H was enhanced with the reduced pore size and increased Ca^2+^ concentration. As a result, the creep of alite paste was improved in the presence of CNTs. Moreover, building on the “ion‐water interlocking mechanism” of cement cohesion proposed by Goyal et al.,^[^ ^53^ ^]^ the hydration of alite generated a high concentration of OH⁻ ions, rendering the surface of the formed C‐S‐H negatively charged. The incorporation of CNTs further enhanced the dissolution of alite, confining water molecules and additional Ca^2^⁺ ions within the charged C‐S‐H surfaces. This process amplified the ionic correlation forces, guiding the gradual densification and solidification of C‐S‐H gel pores. This finding also explains how CNT incorporation enhanced cohesion between C‐S‐H layers and contributed to the increase of the HD C‐S‐H ratio.

## 3 Discussion

Due to the exceptional physicomechanical properties of CNTs, extensive research has been conducted on CNT‐cement composites. However, the interaction mechanisms between CNTs and cement remain a topic of intense debate. The prevailing explanations for CNTs enhancing the mechanical properties of cement‐based materials focus on three aspects: nucleation, crack‐bridging, and filling effects. First, these explanations are generalized. Mechanical property enhancements, such as compressive strength, flexural strength, and modulus, should be considered from different perspectives depending on the stress mode. For instance, the crack‐bridging effect is crucial in tensile experiments, while the influence of CNTs on creep behavior is significant for viscoelasticity. Second, these interpretations are one‐sided, often emphasizing the synergistic interaction between CNTs and cement‐based materials while overlooking how CNT addition affects cement dissolution and interactions between hydrated particles. Therefore, it is essential to consider the unique nanogranular characteristics of hardened cement pastes and explore how CNTs influence the formation, accumulation, and slip of nanoparticles. Our study offers a new perspective on these issues, particularly regarding the mechanism by which CNTs affect the elastic and viscoelastic behaviors of cementitious materials.

### 3.1 Early Hydration: Calcium Ion Enrichment and Weak Nucleation

**Figure 6:**
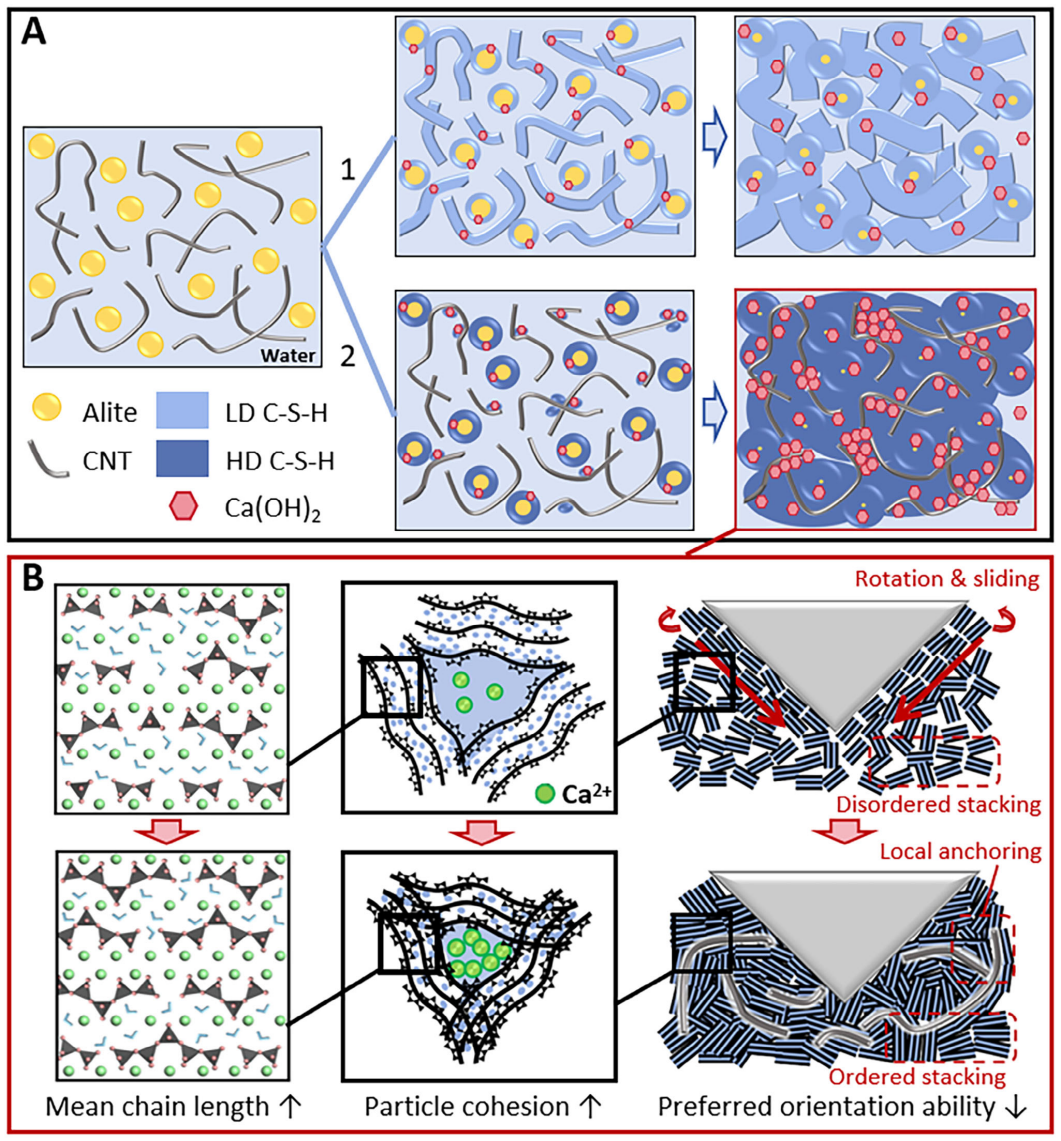
Schematic of the proposed mechanisms for early hydration and creep behavior within alite‐CNT composites. A) Two hypotheses on the mechanism of hydration product formation in the alite with CNTs. B) The influence of CNTs on the degree of polymerization in the C‐S‐H layer, cohesion, and preferred orientation between C‐S‐H layers under loading.

Beginning with the reaction timeline of alite‐CNT composites, we investigated the mechanisms of dissolution and nucleation phases. ICP analysis shows a progressive increase in pore solution Ca^2^⁺ concentration with CNT content (Figure 1A), corroborated by TEM (Figure 1C) and MD simulations (Figure 5A) demonstrating Ca^2^⁺ adsorption onto CNT surfaces via electrostatic interactions. The relatively high pH of the surrounding environment may lead to the physical adsorption of hydroxyl ions onto the CNT surface, further enhancing the adsorption of Ca^2+^ and consequently accelerating the dissolution rate of alite. Next, we summarized two controversial ideas about the nucleation effect (**Figure** **6**A). The first speculation posits that CNTs, utilizing their high specific surface area, act as nucleation sites for C‐S‐H as hydration progresses, leading to dense pastes through layer‐by‐layer nucleation and aggregation on their surfaces.^[^ ^15^, ^54^ ^]^ The second hypothesis suggests that CNTs play only a weak nucleation role, with C‐S‐H primarily using clinker as the nucleation site.^[^ ^27^, ^55^ ^]^ Our findings aligned with the latter mechanism, contradicting the explanation that CNTs' nucleation effect is the primary driver of mechanical property enhancement.^[^ ^15^, ^26^ ^]^ Regarding the role of CNTs during the deceleration period of alite hydration, related studies found that even after 10 h, CNTs still showed no notable nucleation effect.^[^ ^27^ ^]^ Consequently, we hypothesize that CNTs offer minimal nucleation sites even in the later hydration stages, and ultimately, they are primarily compacted alongside the continuously forming hydration products. This is because the clinker exhibits more pronounced advantages as nucleation sites compared to CNTs. First, the content of alite is significantly higher than that of CNTs and is distributed more uniformly. Second, the multi‐walled CNTs we used are chemically inert on their surface, whereas the surface of alite is rich in calcium ions and silicate ions, which lowers the nucleation energy barrier and is more conducive to the nucleation and growth of C‐S‐H.^[^ ^37^ ^]^ However, if CNTs do not strongly seed, how do they induce more HD C‐S‐H formation (Figure 6A)?

### 3.2 Intermediate Structural Evolution: C‐S‐H Nanostructure Refinement

This question has been explored in numerous studies, often emphasizing the “filling effect.”^[^ ^23^, ^29^ ^]^ The explanation is that CNTs reduce the porosity of hardened pastes by filling pores, voids, and cracks within hydration products (Figure 2C). However, another overlooked issue arises: given that CNTs we normally used (e.g., <10 µm in length and 30–50 nm in diameter) are much larger than C‐S‐H particles (≈5 nm), it is difficult for CNTs to fill in between the defective hydration product particles, especially gel pores (Figure 2B). Thus, we shifted focus to the interactions between C‐S‐H particles.

As hydration progresses, CNTs influence C‐S‐H formation and assembly. ICP results show a gradual increase in Ca^2+^ concentration within the pore solution, while NMR results indicate that CNTs enhance the mean chain length of C‐S‐H (Figure 4C). This suggests that elevated calcium ions in the system primarily exist in the pore solution and subsequently participate in the formation of more hydration products like CH (Section 2.2), rather than acting as bridging calcium to affect silicate chain polymerization. TEM at 7 h shows denser C‐S‐H morphology in alite‐CNT groups, and the calculated interlayer spacing is reduced (Figure 1B). In conjunction with MD simulations (Figure 5C), it is confirmed that as C‐S‐H continuously formed and stacked, the increased Ca^2^⁺ ions within the gel pores strengthen the Coulombic attraction between C‐S‐H layers, thereby guiding the dense packing of C‐S‐H from the early hydration stage and facilitating the formation of a greater proportion of HD C‐S‐H.^[^ ^57^, ^58^ ^]^

Notably, the accelerating effect of CNTs on alite hydration is not pronounced (Figure S1, Supporting Information), potentially attributable to several factors: first, the weak nucleation capacity of CNTs failed to provide substantial additional nucleation sites for hydration products; second, the presence of CNTs influenced the spatial formation of hydration products and the ion transport rate; third, the denser hydration products enveloped unhydrated alite particles, thereby retarding further dissolution and precipitation processes.

Additionally, considering that we used the same mass of SP in four sample groups, it plays a significant role in the hydration process. Related studies have shown that SP interacts solely with the Ca‐O layer, without affecting the polymerization degree of silicate chains or intercalating into the C‐S‐H layer.^[^ ^58^ ^]^ Regarding the interaction between CNTs and the pore solution of alite, experiments revealed that the presence of SP further increased the calcium ion content on the CNT surface, promoting the formation of CH.^[^ ^27^ ^]^ Therefore, combined with our experimental results, SP may further regulate calcium ion distribution during hydration through electrostatic adsorption, with its carboxylate groups binding to calcium ions in the pore solution.^[^ ^58^ ^]^ Simultaneously, SP adsorbed onto different phases via its carboxyl groups, ultimately influencing CH formation.^[^ ^27^ ^]^

### 3.3 Long‐Term Properties: Elastic and Viscoelastic Enhancements

Naturally, the development in the early stage controlled the formation of pores and packing mode during the later hardening process (Figures 2 and 4A). AFM confirmed this phenomenon, showing strong cohesive forces after CNT addition (Figure 3G,I). Previous studies have demonstrated the importance of porosity and C‐S‐H cohesion for cementitious materials, particularly the elastic modulus.^[^ ^48^, ^53^ ^]^ This explains the enhancement of the elastic properties of alite paste by CNTs (Figure 3D,E). Certainly, the nanoindentation results reflect the properties of the entire interaction volume (with an affected range approximately three times the indentation depth).^[^ ^48^ ^]^ Although the CNT content is extremely low, their high aspect ratio may facilitate the formation of an overlapping structure within the interaction volume, thereby achieving a localized reinforcement effect. Additionally, we observed that CNTs could increase the degree of polymerization of C‐S‐H at 28 d (Figure 4C), possibly due to the following reasons: first, CNTs enhanced the pH of the solution by accelerating the dissolution of alite, which in turn promoted the deprotonation of silicates, facilitating their polymerization;^[^ ^56^ ^]^ second, the presence of CNTs also constrains nearby Ca^2+^ ions, limiting their interference in the polymerization process of adjacent silicate chains through calcium ion bridging.^[^ ^59^ ^]^

Differing from elastic enhancements, viscoelasticity encompasses dynamic relaxation processes stemming from dynamic resistance to particle rearrangement. For cementitious materials' unique nanogranular characteristics, particle slipping and stacking warrant attention. Figure 6B summarizes the CNTs' mechanism on paste creep behavior. Beyond enhancing intralayer polymerization, increased interlayer cohesion led to a higher packing density of C‐S‐H particles. Relevant simulation studies^[^ ^60^ ^]^ have confirmed that, under the influence of high charge density, C‐S‐H nanoplatelets exhibit a pronounced tendency toward ordered stacking. Furthermore, interparticle cohesion reaches its maximum when adjacent layers are aligned in parallel.^[^ ^61^ ^]^ Also, prior research has demonstrated the impact of nanoplatelet stacking on reorientation.^[^ ^62^, ^63^ ^]^ At lower packing densities (where disordered stacking predominates), externally applied forces induce rotation and sliding of C‐S‐H, resulting in localized rapid preferred orientation and a consequent reduction in mechanical properties. Therefore, building upon the optimization of densely packed C‐S‐H, the presence of CNTs further contributed by functioning as nanofibers. Specifically, they were spatially extruded alongside the HD C‐S‐H phase, mitigating frictional slip caused by free space and instead capitalizing on their anchoring benefits (Figure 3G,H).^[^ ^64^ ^]^

Experimental results also showed that CNT incorporation significantly increased paste creep modulus (Figure 3C). This is mainly due to two factors: first, optimized inter‐particle stacking of C‐S‐H particles enhanced Coulombic forces, improving shear resistance to creep (Figure 5C); second, C‐S‐H interlocking with CNTs may reduce C‐S‐H particles' preferred orientation ability, and the tight backbone‐matrix structure significantly improved integrity. Compared to changes in C‐S‐H layer polymerization degree, C‐S‐H particle stacking and rearrangement are decisive factors in influencing the creep of pastes.^[^ ^65^, ^66^, ^67^ ^]^

It should be noted that in the study of long‐term elastic and viscoelastic enhancements, our focus was on alite's main hydration product (C‐S‐H), with less attention to the relatively small percentage of CH. Although CH has been shown to have little effect on the Young's modulus of C‐S‐H,^[^ ^48^ ^]^ future studies on the overall creep behavior of alite‐CNT composites should also consider the synergistic effect of C‐S‐H and CH.

### 3.4 Reconciling Mechanisms Across Time

The apparent disconnect between the modest early hydration acceleration and pronounced 28‐day mechanical improvements stems from the cumulative nature of CNT effects. While weak nucleation and minimal hydration acceleration suggest limited kinetic influence, the increased Ca^2^⁺ availability and localized adsorption by CNTs initiate subtle but critical changes in C‐S‐H and CH formation. These changes—denser C‐S‐H, longer chains, and higher CH content—compound over 28 days, synergistically affecting both elastic and viscoelastic properties. Thus, the primary mechanism of CNTs lies not in accelerating hydration rates but in modulating ion distributions and gel structure throughout the hydration process.

Note that although our work is based on micro‐scale creep experiments, the method can be upgraded with a homogenization scheme to study long‐term creep prediction of macroscopic nanofiber concrete.^[^ ^68^ ^]^ Therefore, a bottom‐up understanding of CNTs' effect and evolution on cement cohesion from an atomic perspective is key to designing future high‐performance cementitious materials, for example, by designing C‐S‐H interlayer ions and stacking modes to develop construction materials with high elastic‐viscoelastic performance.

## 4 Conclusion

This study redefines the role of CNTs in improving the elastic and viscoelastic properties of alite paste, emphasizing ion‐mediated C‐S‐H refinement, enhanced CH spatial distribution, and optimized nanoplatelet interactions, rather than significant nucleation or filling effects. We observe that CNTs display a limited nucleation effect, with alite acting as the primary nucleation site. Instead, the key mechanism involves CNTs' ability to regulate calcium ion distributions, thereby enhancing the C‐S‐H cohesion and promoting increased CH formation throughout hydration, as evidenced by multiscale indentation and simulations. By optimizing C‐S‐H stacking and reducing slippage, CNTs significantly enhance elastic modulus and creep resistance. These findings underscore that CNT‐induced improvements hinge on sustained ionic and structural modulation, providing a new blueprint for designing advanced nanofiber‐cement composites with superior mechanical performance.

## 5 Experimental Section

### Experimental Design

The alite used in this work was purchased from Kunshan Chinese Technology New Materials Co., Ltd. Figure S6A (Supporting Information) shows the characterization results of the commercial alite. The crystalline phases were analyzed by quantitative X‐ray diffraction (Q‐XRD). The results showed that the purity of alite was ≈92.3 wt.% except for a small amount of β‐C_2_S. The chemical compositions of alite are listed in Table S1 (Supporting Information), which were determined by an X‐ray fluorescence (XRF) spectrometer (Rangaku Corporation). The unfunctionalized multi‐walled carbon nanotube was sourced from Chengdu Organic Chemicals Co. Ltd., and the properties and morphology are shown in Table S2 and Figure S6B (Supporting Information).

In this study, four groups of materials were designed to investigate the mechanisms between CNT and alite (R, T1, T2, and T3). Before mixing, to ensure the uniform dispersion of CNT, a superplasticizer (SP, BASF‐SKY) was selected to prepare the CNT suspension. The dispersion consisted of magnetic stirring for 2 h and ultrasonication for an hour (amplitude 40%, Uibra Cell‐SONICS).^[^ ^42^ ^]^ R, the reference group, consisted of alite, water, and SP at a ratio of (1:0.3:0.004).^[^ ^42^ ^]^ Note that the SP ratio here represented solid content. To evaluate the effects of CNT dosage, T1, T2, and T3 groups were prepared. Among them, the change made based on the reference group was the incorporation of CNT, the content of which was 0.1%, 0.2%, and 0.3% of the mass of alite, respectively. The pastes were cast into 10 mm × 10 mm × 10 mm cubic molds and cured at 23 ± 2 °C and 97% relative humidity for 28 days before the test.

### Hydration Properties

The isothermal conduction calorimeter (Calmetrix‐I‐CAL) was used to monitor the heat release of the pastes for 48 h, and the room temperature was 20 ± 2 °C. Inductively coupled plasma‐optical emission spectrometry (ICP‐OES, SpectroBlue) was used to determine the calcium ion concentration at different hydration periods. The preparation method of the four samples was the same, but the water‐to‐alite ratio was changed to 100 to ensure adequate hydration and obtain the pore solutions.^[^ ^69^ ^]^ The mixtures were continuously stirred under the action of a magnetic stirrer and filtered through 0.45 µm nylon filter membranes at 5 min, 1 h, 3 h, 5 h, and 7 h, respectively. Before testing, the filtrates were treated with concentrated nitric acid. A field emission transmission electron microscope (JEOL JEM‐2100F) was used to observe the nucleation sites and ion distributions around CNTs under an acceleration voltage of 200 kV. Corresponding to the age chosen by ICP‐OES, the hydrated pastes at 5 min, 3 h, and 7 h were extracted and immersed in ethanol. Before sample preparation, ultrasonic dispersion was carried out for five minutes, and then the suspension was taken and deposited onto the carbon‐coated copper grid for observation.

### Nitrogen Adsorption and Field Emission Scanning Electron Microscopy

The nitrogen adsorption method was used to investigate the pore structure of pastes (2–200 nm) using the Micromeritics ASAP 2020 apparatus. The 28‐day cured samples were dried and crushed into homogeneous particles. Before the test, particles were inserted in glass tubes preheated at 60 °C and degassed for 24 h. The analysis was carried out based on the apparatus software. The morphologies of CNTs and pastes were captured by using a field emission scanning electron microscope (Tescan MIRA). The samples were pre‐treated by vacuum drying and gold spraying.

### Confocal Raman Microspectroscopy and X‐Ray Diffraction

A confocal Raman microspectroscopy (CRM) system (Renishaw inVia) was used to obtain optical images and Raman spectra ranging from 240 to 1980 cm^−1^. The system was equipped with a charged‐coupled device, an 1800 g mm^−1^ grating, and a 532 nm laser with a maximum excitation power of 50 mW. The excitation wavelength was calibrated by matching the first peak of a silicon wafer to 520 cm^−1^. A 100 × objective (N.A. = 0.75) was used in the air (*n* = 1.0). Raman mapping was initiated right after removing the cover glass from the top of the cured sample to prevent carbonation. A line of laser (i.e., streamline mapping) with an ND filter of 50% was used to prevent laser‐induced sample damage. This method enabled the rapid collection of a large quantity of spectra with XY spatial resolution of ≈1 µm. The spectra were collected on a grid of points on a 200 µm × 200 µm area with a step size of 2.0 µm, taking 2.6 h per sample. Specifically, the samples tested here were standard‐cured for 14 days, with the aim of observing the distribution of multiple phases in the matrix before it reached a dense state, as well as the influence of CNTs on CH growth.

WiRE and MATLAB software were used for data analysis and visualization. The raw spectra were first processed with cosmic ray removal, background subtraction, and smoothing to increase the signal‐to‐noise ratio (SNR). Then, the Raman spectrum was normalized to compare the relative concentration of components between samples. The band area in the range 240–1200 cm^−1^, representing the matrix composed of unreacted clinker and hydration products, was normalized to 1, using the matrix itself as an internal standard. Note that the G and D band regions of CNTs (1200–1800 cm^−1^) were excluded. Normalizing the spectra over the entire range can underestimate the amount of unreacted clinker and hydrate in the interaction volume because the Raman sensitivity of CNTs is much higher than that of other phases.^[^ ^70^, ^71^ ^]^ The Raman spectrum of C_3_S‐CNT paste was generally a superposition of unreacted C_3_S, C‐S‐H, Ca(OH)_2_, and CNT (Figure S3A, Supporting Information). To describe the data set as a non‐negative combination of these major components, the spectrum was demixed to obtain the independent basis spectra (Figure S3B, Supporting Information). Here, deconvolution analysis on Q_n_ structures was not performed individually, as the focus was on the distribution of phases within the paste. Then, each spectrum was described as the combination of the set of bases by minimizing the error during the curve fitting. The binary phase images are obtained with a threshold of 5 times the background noise to remove the false positives. The SNR of a phase was determined from:

(1) $$\mathrm{SNR}=\left[\mathrm{Intensity}\left(\mathrm{phase}\right)-\mathrm{Mean}\left(\mathrm{background}\right)\right]/\Delta \left(\mathrm{background}\right)$$

where Intensity(phase) refers to the intensity of the characteristic band of a specific phase, and Mean(background) represents the average intensity of a region where no characteristic band was found (1780–1980 cm^−1^). Δ(background) is the standard deviation of the intensity of the same range.

An X‐ray diffractometer (Rigaku SmartLab 9 kW) was employed to identify the hydration degree of the pastes. The measurement condition was under 40 kV and 30 mA. The 2θ scan range was 5°–70° with a step size of 0.02° (0.5 s per step). For quantitative analysis, 20 wt.% α‐Al_2_O_3_ was mixed with each sample as an internal standard. Rietveld refinement was conducted to quantify the phase compositions using the HighScore Plus software. The ICSD codes used for analysis were: C_3_S (4331), C_2_S (81096), Ca(OH)_2_ (202220), and Al_2_O_3_ (164617).

### Indentation and Atomic Force Microscopy

Two kinds of indentation experiments: microindentation tests and nanoindentation tests were performed. The creep performance was investigated using the microindentation test (Bruker Hysitron TI980, Berkovich tip). For each sample, two different regions were selected, and each region was set up as a 4 × 4 dot matrix with a spacing of 300 µm. The loading method was set as loading (10s)‐holding (180s)‐unloading (10s) with a peak load of 1.5 N. Based on the holding stage, the contact creep function at each indentation dot can be expressed as^:[^ ^72^ ^]^

(2) $$L\left(t\right)=L\left(0\right)+\frac{2r_{a}\Delta h\left(t\right)}{P_{m}}=\frac{1}{M_{0}}+\frac{2r_{a}\Delta h\left(t\right)}{P_{m}}$$

where *L*(0) refers to the creep compliance at the initial phase of the holding stage, which can also be represented as 1/*M*_0_; *P_m_* denotes the peak load; *r_a_* is the equivalent radius of the indentation area; Δ*h*(*t*) represents the indentation depth corresponding to the load holding time, which can also be fitted with the logarithmic creep equation.^[^ ^44^ ^]^

In addition, the creep response of C‐S‐H can also be described by the following function:^[^ ^73^ ^]^

(3) $$L\left(t\right)=L\left(0\right)+\frac{1}{C}\ln\left(1+\frac{t}{\tau }\right)$$

where *C* represents the creep modulus; τ denotes the characteristic time. After integrating Equations (2) and (3), the creep modulus can be expressed as:^[^ ^74^ ^]^

(4) $$C=\frac{P_{m}}{2r_{a}a}$$

The elastic modulus and packing density of C‐S‐H were investigated using the nanoindentation test (Bruker Hysitron TI Premier, Berkovich tip). For each sample, four different regions were selected, and each region was set up as a 10 × 10 dot matrix with a spacing of 10 µm. The loading method was set as loading (10s)‐holding (5s)‐unloading (10s) with a peak load of 2000 µN. Based on the unloading stage, the elastic modulus can be calculated for each indentation dot:^[^ ^75^ ^]^

(5) $$\frac{1}{E_{r}}=\frac{1-\vartheta^{2}}{E_{p}}+\frac{1-\vartheta_{i}^{2}}{E_{i}}$$

where *E_r_* and *E_p_* are the reduced modulus and elastic modulus of the samples; *E_i_* is the modulus of the indenter (*E_i_* = 1140 GPa); ϑ and ϑ_*i*_ are the Poisson's ratios of the samples and indenter (ϑ_*i*_ = 0.07).^[^ ^76^ ^]^ Then, the Gaussian Mixture Model was employed to perform cluster analysis on the nanoindentation results, emphasizing the phase proportions of hydration products.^[^ ^77^ ^]^ Besides, based on the method proposed by Ulm and Fang et al.,^[^ ^78^, ^79^ ^]^ the packing density was calculated.

The cohesion of C‐S‐H was further studied using atomic force microscopy (AFM, Bruker Dimension Icon) with standard silicon probes. The morphologies of the samples were first taken by the scanning probe at random locations (scan rate: 0.8 Hz). The force‐displacement curves were tested 100 times (four 5  × 5 dot matrices) for each sample, with a spacing of more than 40 nm. Based on the obtained curves, two parameters was calculated. The adhesion force, also called pull‐off force, can be obtained from the retract curve.^[^ ^80^ ^]^ Based on the method proposed by Lomboy et al.,^[^ ^48^ ^]^ the Hamaker constant A_12_ was calculated to quantify the interactions between C‐S‐H particles.

The preparation of the indentation and AFM samples included drying and embedding with epoxy. After hardening, the samples were polished with 400, 800, 1200, 2000, and 5000 grit polishing papers in sequence, and then polished using finer polishing agents with particle sizes of 9, 3, and 0.05 µm. The samples were ultrasonically cleaned in ethanol between each step, and the duration of each polishing grade was set to 10 min.

### Nuclear Magnetic Resonance Spectroscopy

To investigate the variations of gel pore size among four samples, the measurements were conducted using a ^1^H NMR spectrometer (MesoMR12‐060H‐I), and the resonance frequency was 21.3 MHz (0.5 T). After getting the initial data, the Carr‐Purcell‐Melboom‐Gill method was used to obtain the distribution of T_2_ relaxation times quantitatively. Then, combined with an inverse‐Laplace transform algorithm, the quasi‐continuous T_2_ distributions were achieved.^[^ ^81^ ^]^

To study the polymerization degree of C‐S‐H, ^29^Si nuclei of the powdered samples were scanned using a solid‐state NMR spectrometer (JEOL‐ECZ‐500 MHz). The spinning speed was 4 kHz, and more than 1500 scans were conducted for each sample. Data analysis involved deconvolution of the resulting spectra and then calculating the proportions of Q_n_ and the mean chain length.^[^ ^82^ ^]^ Before the tests, all samples were dried in a vacuum oven for 24 h.

### Molecular Dynamics Simulation

Two simulations were conducted using LAMMPS (http://lammps.sandia.gov).^[^ ^83^ ^]^ For the first simulation, the effect of CNT on calcium ions during alite hydration was investigated. Two layers of CNT were chosen to represent multi‐walled CNT with a length of 98.4 Å, inner diameter and outer diameter of 8.14 and 17.63 Å, respectively (Figure S7A, Supporting Information). H atoms were capped at the end of the CNT to avoid the unsaturated boundary effect. To align with the experiment, the CNT surface here has no functional groups. The solution was modeled with reference to previous studies.^[^ ^84^, ^85^ ^]^ Considering the initial stage of alite dissolution, the Ca/Si ratio of the solution was set to 1.7 (4862 Ca(OH)_2_ and 2860 Si(OH)_4_), and the final size was 100.8 ×  100.8 × 111.0 Å^3^. Subsequently, vacuum layers were built along the Z‐direction on both sides of the solution to ensure a 50 Å layer on the top, and a 15 Å layer on the bottom after the CNT was inserted to prevent unnecessary influence during relaxation.

To accurately model the interactions between the two materials involved, the CVFF and ClayFF force fields (Table S3 and S4, Supporting Information) were employed.^[^ ^86^, ^87^ ^]^ CVFF was utilized to describe the interatomic interactions within the CNT, while ClayFF was applied to the interactions within the solution. To characterize the interactions between the CNT and the solution, arithmetic and geometric mean values were used.^[^ ^88^ ^]^ Water was modeled using the flexible single‐point charge (SPC) model. Following an initial energy minimization process, the isothermal‐isobaric (NPT) ensemble was employed to relax the atomic structures separately for 1 ns, utilizing the Nose‐Hoover thermostat at 300 K and the barostat algorithm at 1 atm.^[^ ^89^, ^90^ ^]^ Subsequently, the isothermal‐isochoric (NVT) ensemble was used for an additional 1 ns to further relax the structures. Note that for the second model containing the CNT, after the rigid CNT was dipped into the solution at a speed of 1 Å ps^−1^, the NPT and NVT ensembles were further used until equilibrium. After obtaining the equilibrated models, calculations under NVT for 1 ns were performed to obtain ion trajectories. All models were set to periodic boundary conditions in three directions with a fixed timestep of 1 fs and a cutoff distance of 12 Å.

For the second simulation, the effects of gel pore sizes and calcium ion concentrations on the C‐S‐H creep behaviors were investigated. The C‐S‐H model construction was based on the method proposed by Pellenq et al.^[^ ^91^ ^]^ A tobermorite structure with an interlayer spacing of 11 Å was used as the initial configuration. After water removal, orthogonal transformation, and deletion of some neutral SiO_2_ and Si_2_O_5_ groups, the Grand Canonical Monte Carlo water absorption simulation was performed until the final unit model containing two C‐S‐H layers was obtained. To simulate the shear between C‐S‐H particles, a supercell process was first performed on the unit cell (3 × 2 × 1) (Figure S7B, Supporting Information). Then, a 30 Å vacuum space was created between two C‐S‐H layers to ensure stable relaxation after the subsequent insertion of solutions. The solutions here consisted of two types: 20 Å and 10 Å thicknesses, corresponding to simulated gel pores of different sizes.^[^ ^92^, ^93^ ^]^ The density of the solutions was fixed at 1 g cm^−3^. According to the experimental results in Figure 1A and other related studies,^[^ ^65^, ^94^ ^]^ due to the low concentration of calcium ions in the pore solution, six and thirty calcium ions were mixed into the solution of 20 and 10 Å for better comparison. Next, the two solutions were placed in the middle of the interlayer space. The final two models contained 5547 and 4086 atoms, respectively.

Using the ClayFF force field, the parameters for describing the interatomic interactions were taken from Cygan et al.^[^ ^87^ ^]^ These interactions include bonds and angles, van der Waals interactions, and Coulomb interactions. Water was considered using the SPC model. After the initial energy minimization process, the NPT ensemble was used to relax the atomic structures for 2 ns using the Nose‐Hoover thermostat at 300 K and the barostat algorithm at 1 atm. After obtaining the equilibrated models, a shear strain was applied in the xz plane with steps of 0.01%/ps. All models were set to periodic boundary conditions in three directions with a fixed timestep of 1 fs and a cutoff distance of 12 Å.

## Conflict of Interest

The authors declare no conflict of interest.

## Author Contributions

X.C. was responsible for writing the original draft, software, methodology, formal analysis, and investigation; J.P. contributed to methodology and formal analysis; W.J. participated in formal analysis; Y.H. assisted with methodology; J.‐X.L. provided supervision, conceptualization, and writing – review and editing; Z.H. contributed to conceptualization and writing – review and editing; and C.‐S.P. handled validation, writing – review and editing, funding acquisition, and project administration.

## Supporting information



Supporting Information

## Data Availability

The data that support the findings of this study are available from the corresponding author upon reasonable request.
