# Supplementary material for: Unlocking the Interaction Mechanism of CNTs and C‐S‐H on Enhancing Elastic and Viscoelastic Properties of Alite Paste
Source: Adv Sci (Weinh). 2025 Jul 17;12(38):e05876. doi: 10.1002/advs.202505876 (PMC12520525; doi:10.1002/advs.202505876)
Supplement: Supplementary file 1 — Supporting Information [file ADVS-12-e05876-s001.docx]

Supporting Information

Unlocking the Interaction Mechanism of CNTs and C-S-H on Enhancing Elastic and Viscoelastic Properties of Alite Paste

Xi Chen, Jiseul Park, Weiyi Ji, Yujie Huang, Jian-Xin Lu*, Zhangli Hu, Chi Sun Poon*


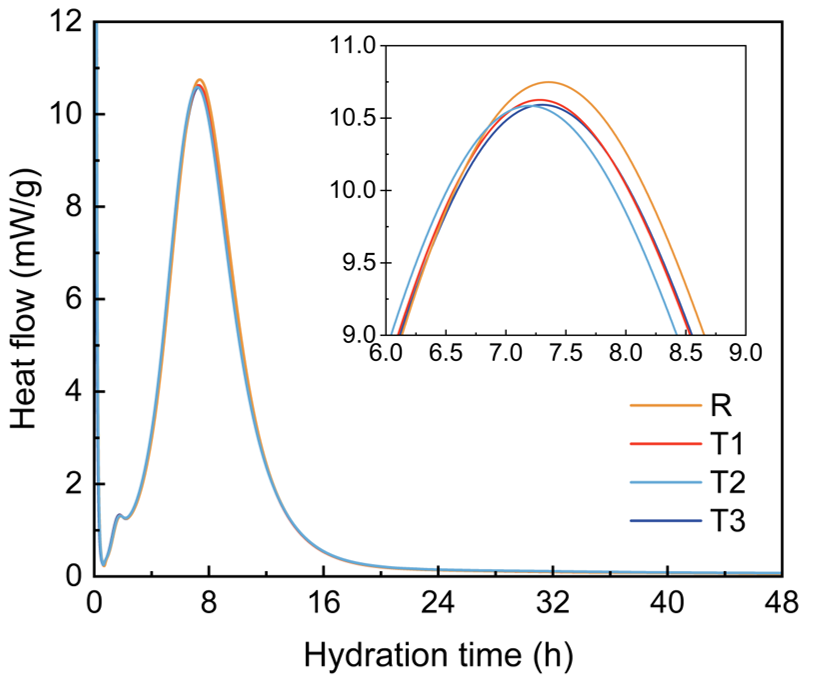


**Figure S1. Heat flow curves of alite pastes with different dosages of CNTs**. The peaks are shown at higher magnification in the upper right corner.


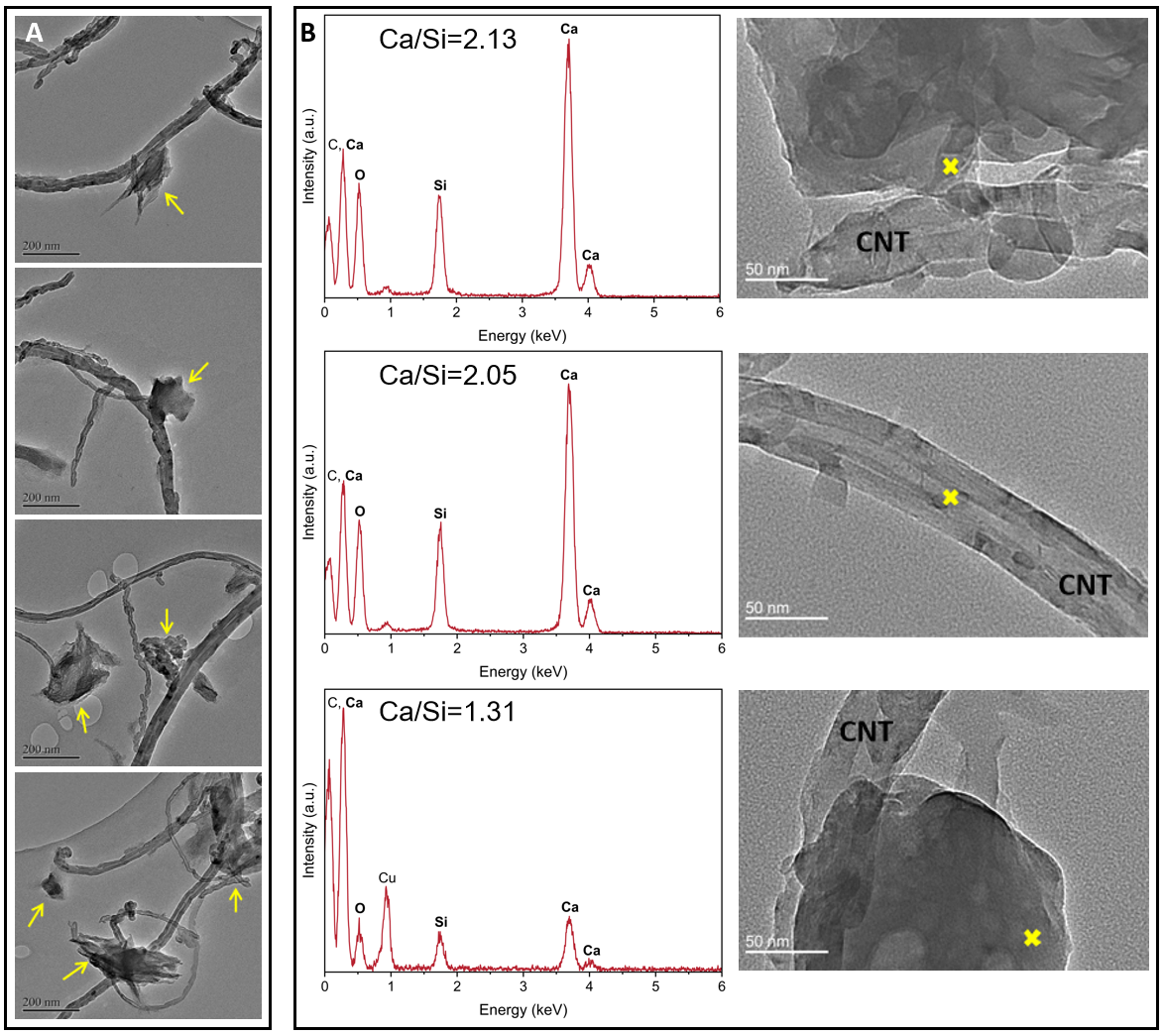


**Figure S2. TEM analysis of nucleation sites. (A)** Nucleation sites observed on the surface of CNTs. The time to terminate hydration was 7 hours after the beginning of alite dissolution, and a few nucleation sites can be observed. **(B)** Multi-point elemental analysis of nucleation sites around CNTs.


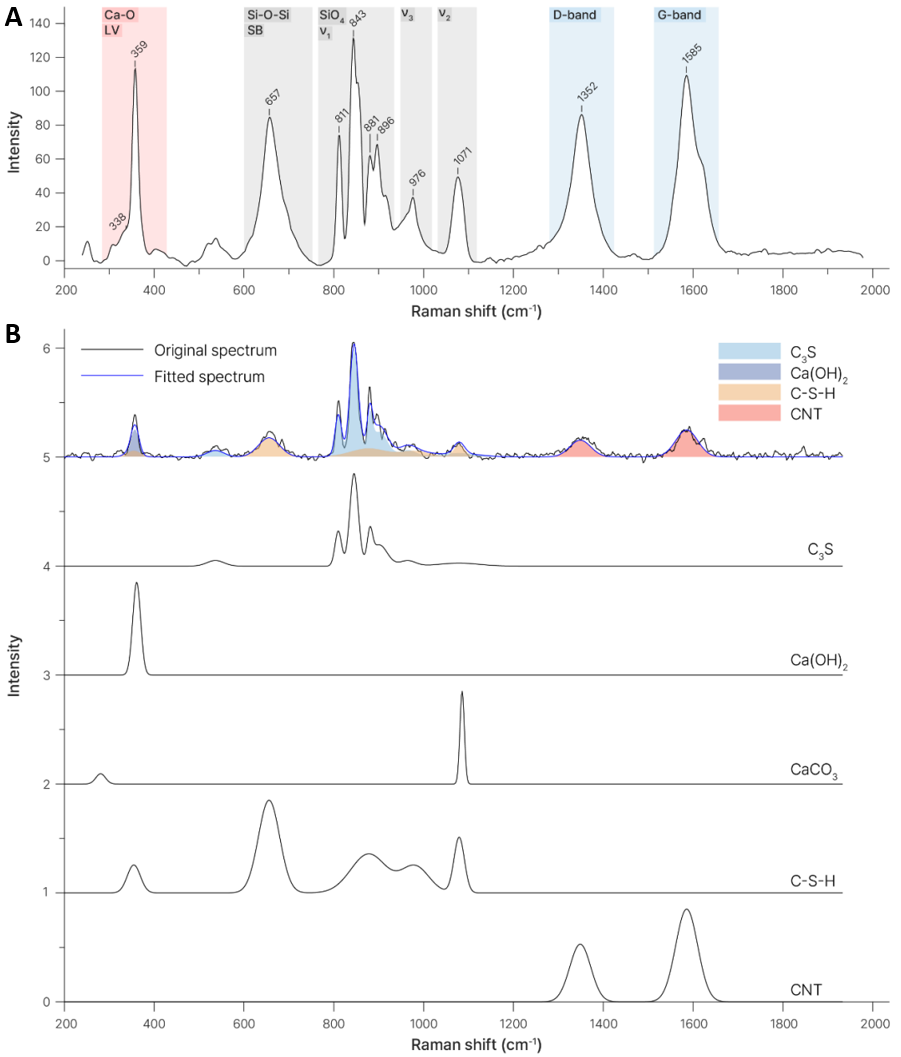


**Figure S3. The processing of Raman spectrum. (A)** Average Raman spectrum of alite-CNT composites containing 0.2% CNTs in 200 µm × 200 µm area, with peak positions and vibrational modes labeled. **(B)** An example of fitting result at specific location of T2.


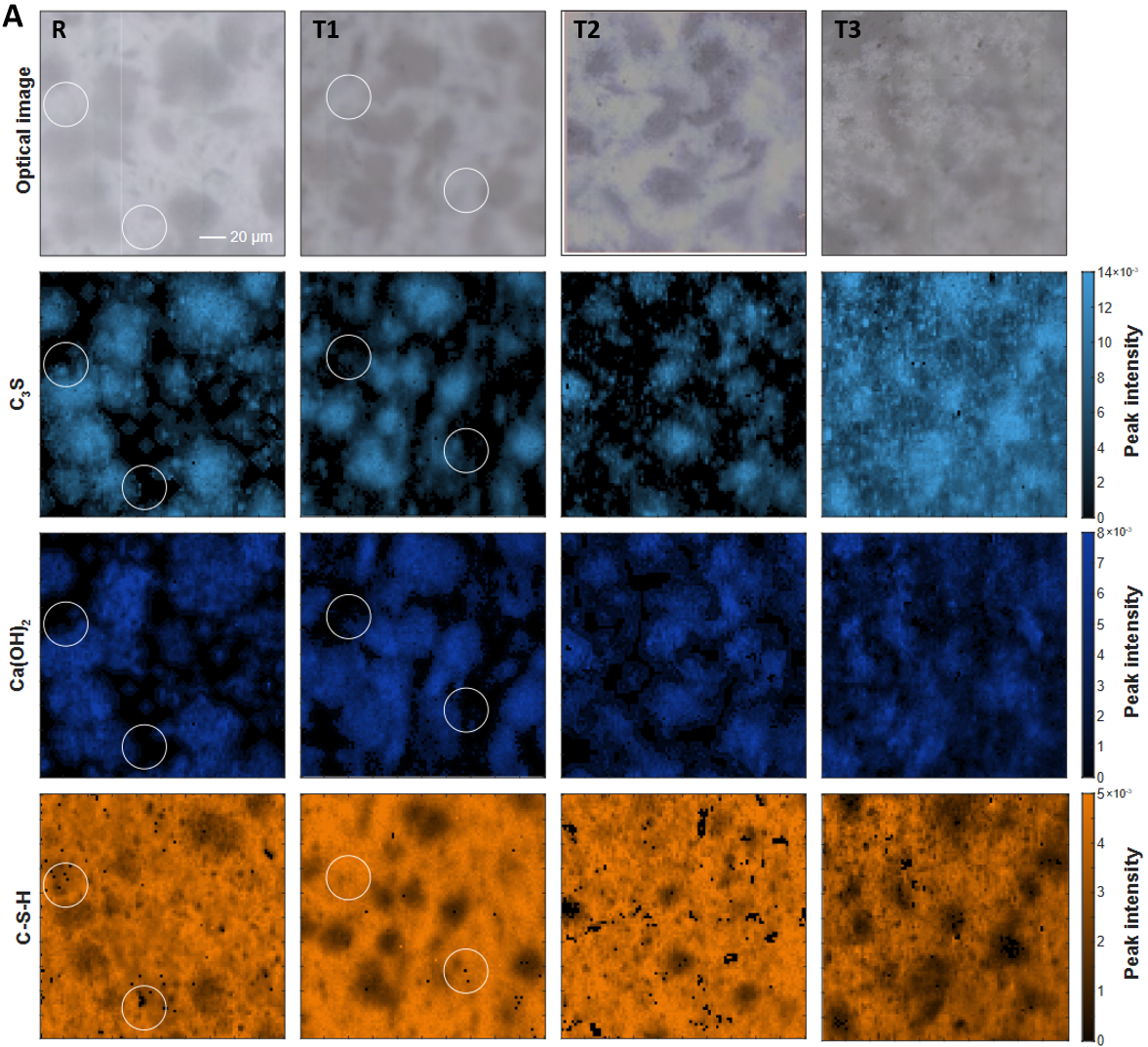


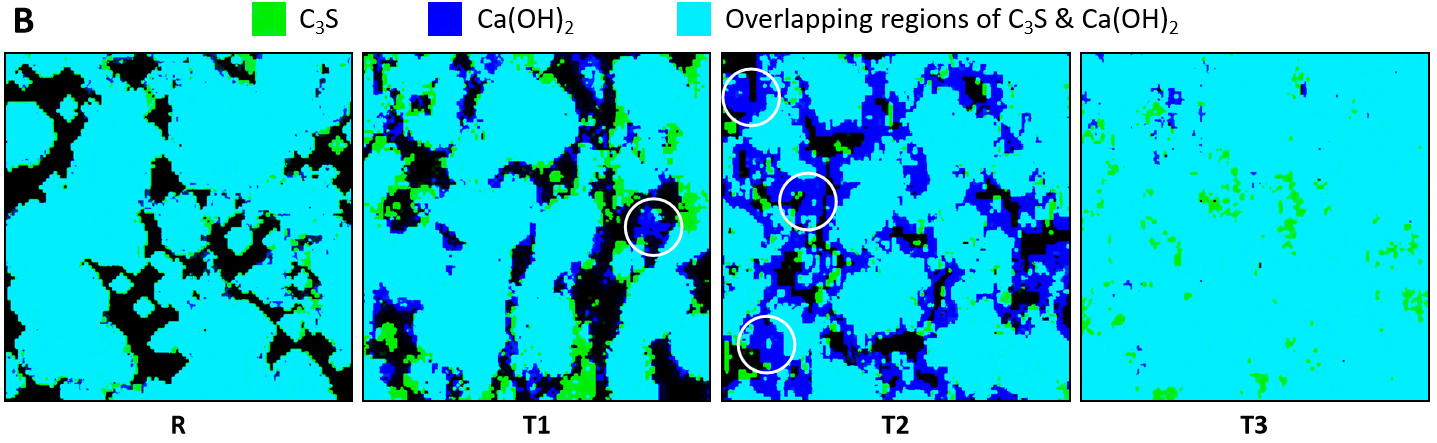


**Figure S4. Results of Confocal Raman Microspectroscopy. (A)** Optical images and spatial distribution maps of C_3_S, Ca(OH)_2_, and C-S-H in alite-CNT composites with different CNT contents. **(B)** Comparison of CNT effects on Ca(OH)_2_ distribution across four groups.





**Figure S5. Quantitative statistics of the proportions of LD C-S-H and HD C-S-H based on the combined results of XRD and nanoindentation.**

**
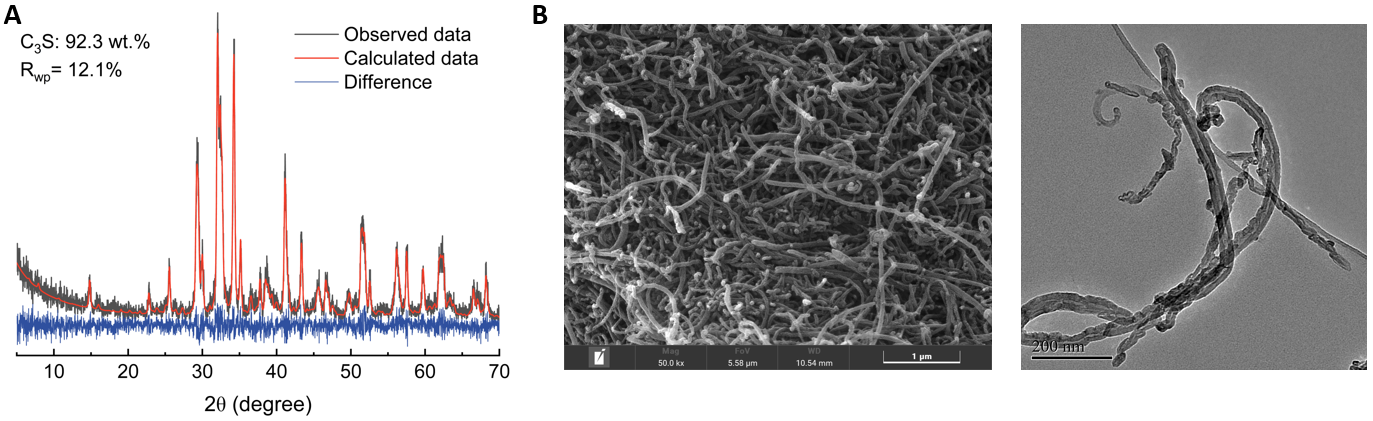
**

**Figure S6. Experimental material characterization. (A)** Crystalline phase composition of alite by Rietveld analysis. **(B)** Morphology of multi-walled carbon nanotubes.

**Table S1.** Chemical compositions of alite.

| Composition | CaO | SiO_2_ | MgO | Al_2_O_3_ | P_2_O_5_ | K_2_O | SrO | Cl |
| --- | --- | --- | --- | --- | --- | --- | --- | --- |
| wt. % | 80.2 | 19.4 | 0.111 | 0.0849 | 0.116 | 0.0349 | 0.0176 | 0.0093 |

**Table S2.** Properties of multi-walled carbon nanotubes.

| Property | Length (μm) | Inner diameter (nm) | Outer diameter (nm) | Purity  (wt. %) | Specific surface area (m^2^/g) | Density (g/cm^3^) |
| --- | --- | --- | --- | --- | --- | --- |
| MWCNT | ＜10 | 5-15 | 30-50 | ＞98 % | ＞100 | 2.1 |

**
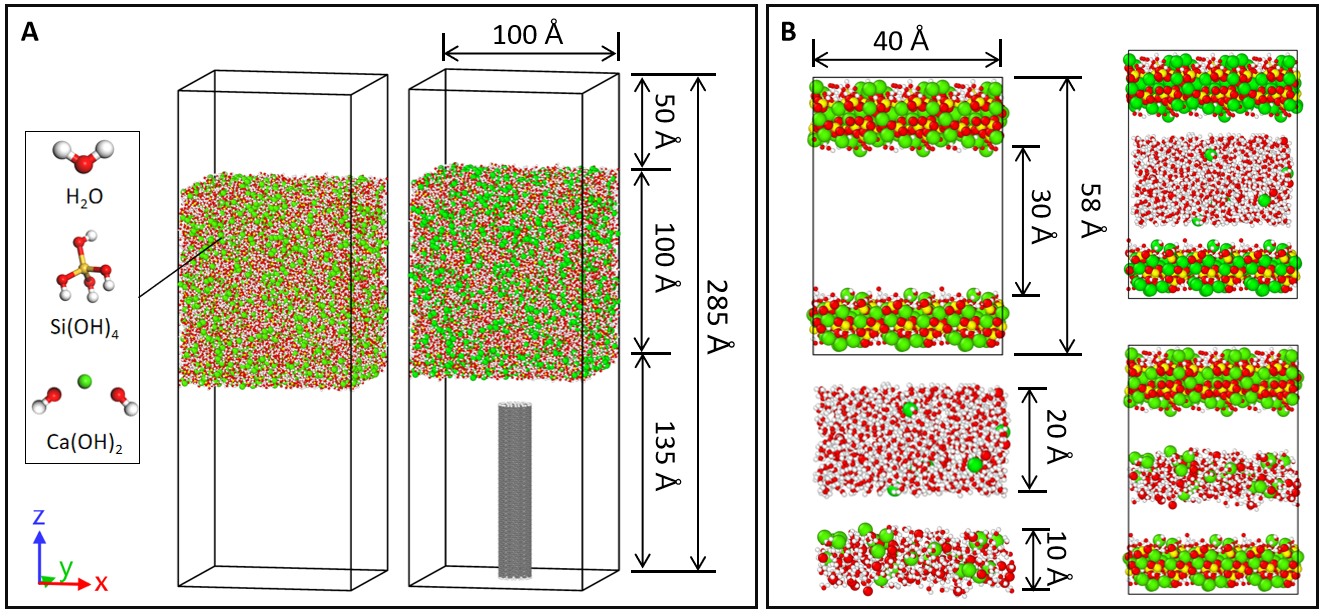
**

**Figure S7. Modeling process of molecular dynamics simulation. (A)** Pore solution models without CNT and with CNT. **(B)** Shear models with different pore sizes and ion concentrations. Calcium (green), silicon (yellow), oxygen (red), hydrogen (white), and carbon (gray).

**Simulation Parameters**

In ClayFF force field, the overall energy is anticipated to comprise contributions from Coulombic (electrostatic) interactions, short-range interactions (commonly known as van der Waals interactions), and bonded interactions (Table S3):

$E_{total}=E_{Coul}+E_{VDW}+E_{bond stretch}+E_{angle bend}$ (1)

$E_{Coul}=\frac{e^{2}}{4\pi\epsilon_{0}}\sum_{i\neq j} \frac{q_{i}q_{j}}{r_{ij}}$ (2)

where $e$ is the charge of the electron, $\epsilon_{0}$ is the dielectric permittivity of vacuum (8.85419×10^-12^ F/m), $q_{i}$ and $q_{j}$ are the partial charges, and $r_{ij}$ is the distance of separation.

$E_{VDW}=\sum_{i\neq j} D_{0,ij}\left[ \left( \frac{R_{0,ij}}{r_{ij}} \right)^{12}-2\left( \frac{R_{0,ij}}{r_{ij}} \right)^{6} \right]$ (3)

$R_{0,ij}=\frac{1}{2}(R_{0,i}+R_{0,j})$ (4)

$D_{0,ij}=\sqrt{D_{0,i}D_{0,j}}$ (5)

where $R_{0,ij}$ and $D_{0,ij}$ are the interaction parameters between the different atoms and are calculated according to the arithmetic mean rule for the distance parameter, $R_{0}$, and the geometric mean rule for the energy parameter, $D_{0}$.

$E_{bond stretch ij}=k_{1}\left( r_{ij}-r_{0} \right)^{2}$ (6)

$E_{angle bend ijk}=k_{2}\left( \theta_{ijk}-\theta_{0} \right)^{2}$ (7)

where $k_{1}$ and $k_{2}$ are force constants, $r_{0}$ and $\theta_{0}$ represent the equilibrium bond length and bond angle, respectively.

**Table S3**. Parameters for the CLAYFF Force Field.

| **Nonbond Parameters** | | | | |
| --- | --- | --- | --- | --- |
| species | | symbol | D_0_ (kcal/mol) | R_0_ (Å) |
| water hydrogen | | h* |  |  |
| hydroxyl hydrogen | | ho |  |  |
| water oxygen | | o* | 0.1554 | 3.5532 |
| hydroxyl oxygen | | oh | 0.1554 | 3.5532 |
| bridging oxygen | | ob | 0.1554 | 3.5532 |
| tetrahedral silicon | | st | 1.8405×10-6 | 3.7064 |
| hydroxide calcium | | cah | 5.0298×10-6 | 6.2428 |
| aqueous calcium ion | | Ca | 0.1000 | 3.2237 |
| **Bond Parameters** | | | | |
| bond stretch | | | k_1_ (kcal/mol Å^2^) | r_0_ (Å) |
| species i | species j | |  |  |
| o* | h* | | 554.1349 | 1.0000 |
| oh | ho | | 554.1349 | 1.0000 |
| angle bend | | | k_2_ (kcal/mol rad^2^) | θ_0_ (deg) |
| species i | species j | species k |  |  |
| h* | o* | h* | 45.7696 | 109.47 |

Similarly, in the CVFF force field, the interactions include the following (Table S4):

$E_{VDW}=4\sum_{i\neq j} \varepsilon_{ij}\left[ \left( \frac{\sigma_{ij}}{r_{ij}} \right)^{12}-\left( \frac{\sigma_{ij}}{r_{ij}} \right)^{6} \right]$ (8)

where $\varepsilon_{ij}$ and $\sigma_{ij}$ are the energy well depth and equilibrium distance for the atomic pair.

$E_{bond stretch ij}=k_{1}\left( r_{ij}-r_{0} \right)^{2}$ (9)

$E_{angle bend ijk}=k_{2}\left( \theta_{ijk}-\theta_{0} \right)^{2}$ (10)

$E_{dihedral bend ijkl}=k_{3}\left[ 1+cos\left( n\varphi\right) \right]$ (11)

where $k_{3}$ and $\varphi$ are the tortional stiffness and dihedral.

**Table S4**. Parameters for the CVFF Force Field.

| **Nonbond Parameters** | | | | | | |
| --- | --- | --- | --- | --- | --- | --- |
| species | | symbol | ε_ij_ (kcal/mol) | | σ_ij_ (Å) | |
| sp2 aromatic carbon | | c | 0.074 | | 3.6170 | |
| hydrogen bonded to C | | h | 0.019 | | 2.4500 | |
| **Bond Parameters** | | | | | | |
| bond stretch | | | k_1_ (kcal/mol Å^2^) | | r_0_ (Å) | |
| species i | species j | |  |  |  |  |
| c | c | | 480.0000 | | 1.3400 | |
| c | h | | 363.4164 | | 1.0800 | |
| angle bend | | | k_2_ (kcal/mol rad^2^) | | θ_0_ (deg) | |
| species i | species j | species k |  |  |  |  |
| c | c | c | 90.0000 | | 120.0000 | |
| c | c | h | 37.0000 | | 120.0000 | |
| dihedral bend | | | | k_3_ (kcal/mol) | d | n |
| species i | species j | species k | species l |  |  |  |
| - | c | c | - | 3.0000 | 1 | 2 |
